# Role of adenovirus-mediated retinoblastoma 94 in the treatment of human non-small cell lung cancer

**DOI:** 10.3892/mmr.2015.3227

**Published:** 2015-01-20

**Authors:** FANG LIU, QINGBAO LI, PING ZHANG, FANG CHEN, YUFENG CHENG

**Affiliations:** 1 Department of Radiation Oncology, Qilu Hospital of Shandong University, Jinan, Shandong 250012, P.R. China; 2 Department of Image, Shandong Medical College, Jinan, Shandong 250002, P.R. China; 3 Department of Cardiac Surgery, Shandong Provincial Hospital Affiliated to Shandong University, Jinan, Shandong 250021, P.R. China; 4 Department of Oncology, The People’s Hospital of Binzhou City, Binzhou, Shandong 256610, P.R. China; 5 Department of Ultrasonography, The Second Hospital Affiliated to Shandong University of Traditional Chinese Medicine, Jinan, Shandong 250001, P.R. China

**Keywords:** non-small cell lung cancer, retinoblastoma gene, gene therapy, cell cycle arrest

## Abstract

Non-small cell lung cancer (NSCLC) remains the leading cause of cancer-related mortality despite the fact that great advances have been made in therapeutic treatment methods. Therefore, in the present study, the role of adenovirus-mediated retinoblastoma 94 (Ad-RB94) gene therapy in NSCLC was investigated. Following treatment with Ad-RB94, the proportion of A549 cells in the G2/M phase was increased. In the mouse xenograft model, the overexpression of RB94 inhibited the tumor growth compared with the control group and the Ad*-*LacZ-treated group. In the transplanted tumors, the overexpression of RB94 induced the apoptosis of tumors as well as an increase in the mRNA levels of cyclinB1. In conclusion, the results of the present study suggested that RB94 may effectively inhibit NSCLC tumor cell growth by inducing G2/M cell cycle arrest and apoptosis, indicating that RB94 may be a promising candidate for adjuvant therapy with radiation or chemotherapy in NSCLC.

## Introduction

Non-small cell lung cancer (NSCLC) remains the leading cause of cancer-related mortality in China despite the fact that great advances have been made in the therapeutic treatment methods, including surgical resection, chemotherapy and radiotherapy (1). NSCLC mainly comprises two types: adenocarcinoma and squamous cell carcinoma. Two-thirds of NSCLC patients are diagnosed at a late stage of the disease when the tumors are not resectable. In addition, up to 60% of all resected patients relapse and succumb to the disease (2). Although radiotherapy has been widely used to treat unresectable and metastatic tumors, radio resistance usually leads to a low overall 5-year survival rate of less than 15% (3). Therefore, the development of new treatment options including gene therapy is an urgent requirement.

Gene therapy is one of the innovative approaches which have offered new hope for enhancing antitumor effects. The retinoblastoma (RB94) tumor suppressor gene, which lacks the NH2-terminal 112 amino acid residues of the full-length RB protein (RB110), has been identified to have marked tumor suppressor efficacy compared with wild-type RB110 (4,5). The RB94 protein has been reported to remain in a hypo-phosphorylated state in transfected tumor cells and demonstrate a significantly longer half-life than the RB110 protein (4,5). In addition, RB94 exhibits an increased antitumor efficacy in RB-negative and RB-positive human tumors, including fibrosarcoma and bladder, lung and prostate carcinoma, while the effects of RB110 are limited to RB-negative tumors (4). Preclinical studies also demonstrate that adenovirus-mediated (Ad)-RB94 gene transfer significantly suppresses the growth of human head and neck cancer, bladder carcinoma, pancreatic carcinoma and esophageal cancer *in vitro* and *in vivo* (6–9). Moreover, the combination of RB94 and radiation therapy (XRT) results in synergistic tumor growth suppression of head and neck squamous cell carcinoma (10). Therefore, RB94 appears to be a promising candidate for gene therapy in NSCLC.

In the present study, an adenoviral vector carrying the RB94 gene (Ad-RB94) was constructed. Subsequently, we investigated whether Ad-RB94 exerted therapeutic effects in NSCLC as well as examining the underlying molecular mechanisms.

## Materials and methods

### Cell culture

The human NSCLC cell line A549 has been well characterized and is known to express wild-type RB (11). The A549 cells were cultured in RPMI 1640 (Hyclone, Logan, UT, USA) with 10% fetal bovine serum at 37°C in 5% CO_2_.

### Construction of recombinant adenoviral vectors

The construction of recombinant adenovirus vector for the RB94 gene was carried out using Gateway™ clone technology (GeneSil, Wuhan, China). Briefly, total RNA was extracted from a human embryo and reversed transcribed to obtain object cDNA, then the hRB94 gene fragment was amplified by polymerase chain reaction (PCR). The attB-flanked PCR primers were designed and used to amplify the hRB94 gene by PCR. An entry clone was performed by a BP recombination reaction with attB-PCR products and donor vector pDONRTM221. Then the entry clone and the target vector Ad/CMV/V5-DEST with attR1 and attR2 sites was recombined *in vivo* to create the expression clone (Ad-hRB94) using an efficient LR recombination reaction. Next, the expression clone was confirmed by PCR and sequencing. Ad-hRB94 was digested with Pac I and transferred into 293A cells to be packaged into adenovirus stock. Ad-hRB94 was amplified by infection of 293A cells and the titer was measured. Ad-LacZ, a replication-defective control adenovirus not carrying the RB94 gene, was obtained from Invitrogen Life Technologies (Carlsbad, CA, USA). The viruses were amplified and plaque-purified. Titers were determined by standard plaque assays.

### Western blot analysis

The cells were lysed by adding 100 μl radioimmunoprecipitation assay lysis buffer [1% Triton X-100, 1% deoxycholate, 0.1% sodium dodecyl sulfate (SDS), 1 mM phenylmethylsulfonyl fluoride] purchased from Beyotime Institute of Biotechnology (Shanghai, China) and then quantitated using a bicinchoninic acid (BCA) assay kit with phosphate-buffered saline (PBS) as a standard (Pierce, Rockford, IL, USA). Equal quantities of protein (50 μg) from the different cells were separated by 10% SDS-polyacrylamide gel electrophoresis. The separated proteins were transferred onto nitrocellulose (GE Healthcare, Buckinghamshire, UK). Non-specific binding sites were blocked using PBS-Tween-20 and 5% non-fat dried milk for 1 h at room temperature. Following blocking, the samples were incubated overnight at 4°C with primary antibody mouse-antihuman RB (BD Biosciences Pharmingen, San Diego, CA, USA), which recognizes both full-length RB and NH2-terminal-truncated RB94 proteins, at a concentration of 1:200 in Tris-buffered saline-Tween-20. Membranes were washed three times for 10 min in a buffer containing PBS and 0.1% Tween-20, and then incubated with an appropriate goat antimouse secondary antibody conjugated to horseradish peroxidase (Amersham Pharmacia Biotech, Piscataway, NJ, USA) applied for 1 h at room temperature. Reactive bands were detected with the ECL chemiluminescence reagent (Amersham Pharmacia Biotech, Freiburg, Germany). β-actin was detected using monoclonal anti-β-actin (Sigma, St. Louis, MO, USA).

### Reverse transcription-quantitative PCR (RT-qPCR)

Total mRNA was extracted from tumors in each of the studied groups with an mRNA isolation kit (Takara, Dalian, China), and 3 μg total RNA was used for cDNA synthesis by SuperScript II reverse transcriptase (Invitrogen) according to the standard instructions. qPCR was performed in a final volume of 20 μl containing 1 μl each cDNA template, 2 μl each 10 nM primer (RB94 F: 5′-GAA TCT GCT TGT CCT CTT AAT CTT AAT CTT CC-3′ and R: 5′-GAA GAT GGT GAT GGG ATT TC-3′; cyclinB1 F: 5′-CAG TCA GAC CAA AAT ACC TAC TGG GT-3′ and R: 5′-ACA CAA ACC AGC TGC AGC ATC TTC TT-3′), and 10 μl SYBR-Green Master mix. Quantification was carried out using the comparative cycle threshold (Ct) method, and water was used as the negative control. Ct values were calculated by determining the cycle number at which the fluorescence exceeded the threshold limit. The average Ct values for the target gene were normalized to an endogenous housekeeping gene encoding 18S rRNA.

### Flow cytometric analysis

Cells were seeded at a density of 1×10^6^ cells/well in six-well tissue culture plates and allowed to adhere overnight. Media were removed and cells were incubated with either Ad-RB94 or Ad-LacZ at a multiplicity of infection of 10, or control with PBS in 5 ml media for 2 h, after which 10 ml media was added. After 24 h, harvested cells were trypsinized, washed with PBS, fixed in suspension in 75% ethanol, and kept at −20°C until analysis. Fixed cells were centrifuged and resuspended in PBS. After adding RNase (1 mg/ml) to the cell suspension, the cells were incubated for 30 min at 37°C, and finally stained with propidium iodide (50 μg/ml) for 1 h at 4°C and then analyzed with FACScan. Cell distribution was analyzed by WinMDI 2.8 software (Joseph Trotter, San Diego, CA, USA).

### Xenograft tumor in vivo

Eighteen six-week-old female BALB/c nude mice were injected subcutaneously into the posterior flank region with 5×10^6^ A549 cells in 100 μl normal saline. After 10 days, skin flaps were raised and tumors were exposed. Then mice were divided into three groups randomly: a control group, an Ad-LacZ group and an Ad-RB94 group (n=6 for each). The tumors were injected intratumorally on day 0, 4, 7 and 14 following the establishment of the groups. The mice were sacrificed and the tumors were measured using calipers on day 21. The volumes were calculated according to the formula: V (mm^3^) = larger diameter (mm) × smaller diameter^2^ (mm^2^) × π/6. Institutional guidelines with regard to animal experimentation and care were followed for the nude mice during the experiment.

### Apoptosis detection by Hoechst staining

The apoptosis of tumors was evaluated by Hoechst staining according to the manufacturer’s instructions. First, paraffin sections were deparaffinized, washed once with PBS for 10 min and stained with Hoechst 33258 (Beyotime Institute of Biotechnology) for 5 min. Then they were given neutral gummi and observed by fluorescent Olympus microscopy. Four hundred cells were counted in four different visual fields for every slide. The percentage of apoptotic positive cells was calculated by counting the positive cells and the total number of cells from every visual field.

### Statistical analysis

All the results are expressed as the means ± standard deviation. Statistical significance was determined using SPSS 17.0 for Windows (SPSS, Inc., Chicago, IL, USA). One-way analysis of variance was performed for multiple comparisons followed by Fisher least significant difference post hoc comparisons. P<0.05 was considered to indicate a statistically significant difference.

## Results

### Expression of RB94 following Ad-RB94 transfection

**Figure 1:**
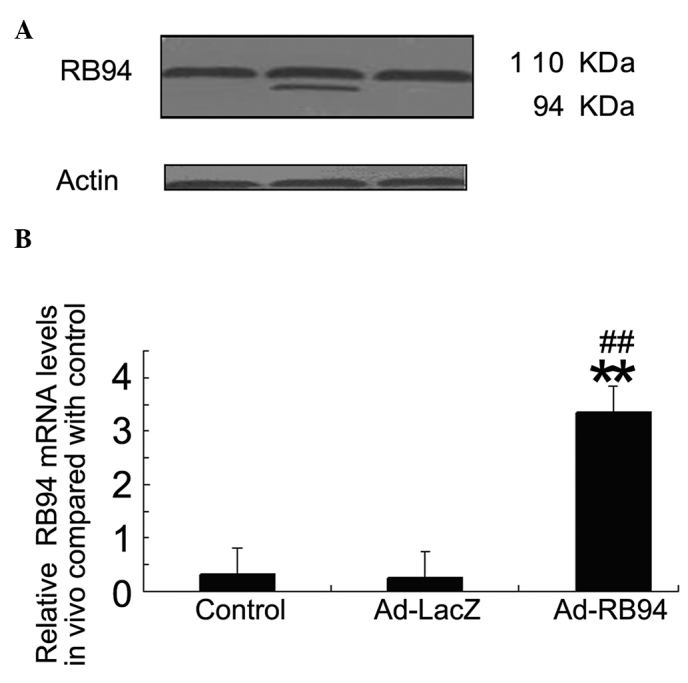
Expression of retinoblastoma (RB) *in vitro* and *in vivo.* (A) Detection of RB94 in A549 cells by western blot analysis. (B) Reverse transcription-quantitative polymerase chain reaction analysis of RB94 mRNA levels *in vivo*. Data are expressed as the means ± SD (n=3). ^**^P<0.01 compared with control; ^##^P<0.01 compared with Ad-LacZ group.

Following Ad-RB94 transfection, western blot analysis revealed two bands at 110 kDa (wild-type RB) and 94 kDa (RB94) in the A549 cells (Fig. 1A) with the RB antibody. However, the control and Ad-LacZ-transfected A549 cells only expressed wild-type RB protein at 110 kDa. Next, the expression of RB94 *in vivo* was determined by RT-qPCR. As shown in Fig. 1B, the *in vivo* RB94 mRNA level was higher in the Ad-RB94 group than in the Ad-LacZ and control groups. There was no significant difference between the control and the Ad-LacZ group.

### Ad-RB94 induces G2/M cell cycle arrest

**Figure 2:**
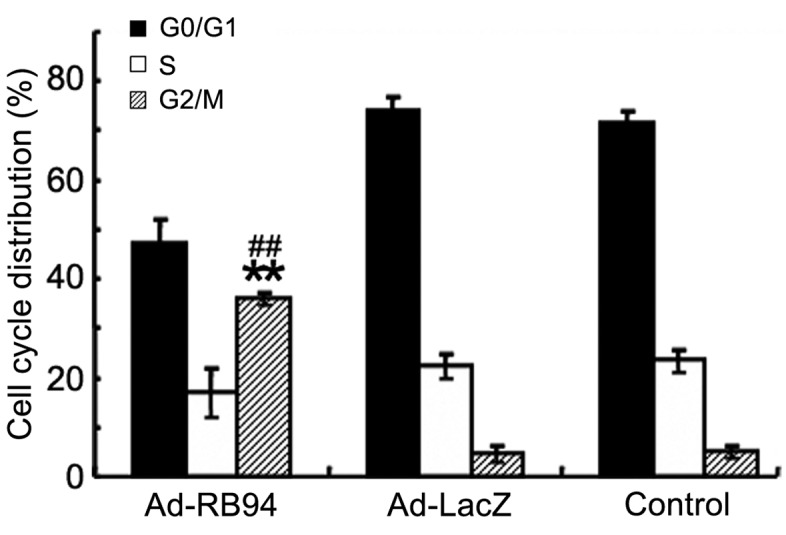
Retinoblastima (RB) 94 gene transfer induces G2/M cell cycle arrest *in vitro*. The experiment was replicated three times. Data are expressed as the means ± SD (n=3). ^**^P<0.01 compared with control; ^##^P<0.01 compared with Ad-LacZ group.

As shown in Fig. 2, flow cytometry revealed that overexpression of Ad-RB94 increased the proportion of A549 cells in the G2/M phase, whereas it decreased the proportion of A549 cells in the G0/G1 phase and the S phase, indicating that Ad-RB94 may induce G2/M cell cycle arrest in A549 cells.

### RB94 transfer inhibits tumors growth and cyclinB1 mRNA levels in vivo

**Figure 3:**
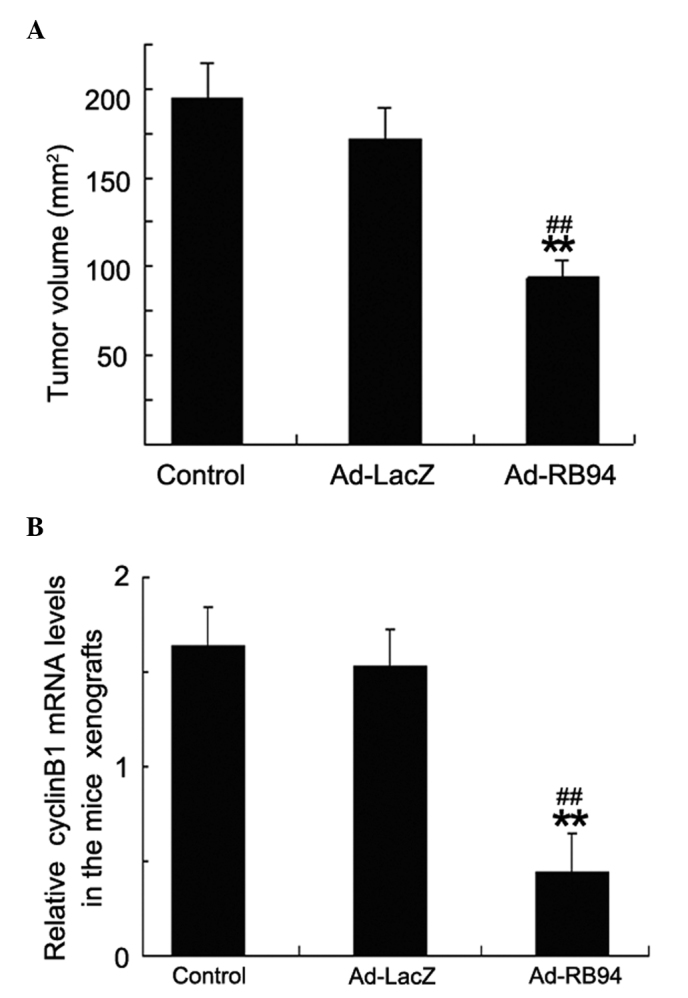
Retinoblastima (RB) 94 transfection suppresses tumor growth *in vivo*. (A) Tumor volumes following treatment. (B) Relative cyclinB1 mRNA in the tumor following RB94 treatment. Data are expressed as the means ± SD (n=6). ^**^P<0.01 compared with control; ^##^P<0.01 compared with Ad-LacZ group.

Following the 21 days of treatment, mice were sacrificed and the volumes of tumors were examined. Treatment with Ad-RB94 significantly inhibited the growth of tumors compared with the control and Ad-LacZ. There was no significant difference between the control and Ad-LacZ groups (P>0.05; Fig. 3A). The cyclinB1 mRNA level of the tumors following Ad-RB94 treatment decreased significantly compared with those of the Ad-LacZ and the control groups (Fig. 3B). There were no significant differences in the levels of cyclinB1 mRNA between the Ad-LacZ and the control groups.

### Apoptosis detection in vivo

**Figure 4:**
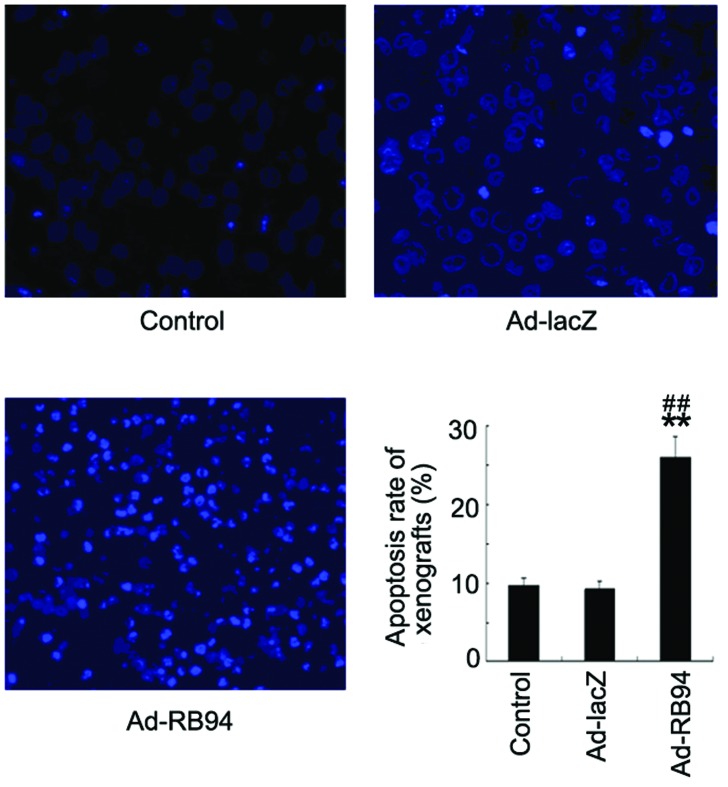
Retinoblastima (RB) 94 transfection induces tumor apoptosis *in vivo*. Data are expressed as the means ± SD (n=6). ^**^P<0.01 compared with control; ^##^P<0.01 compared with Ad-LacZ group.

To further investigate whether Ad-RB94 induced NSCLC apoptosis *in vivo*, an apoptosis detection kit was used to determine apoptosis-related molecular markers in tumor sections (Fig. 4). The results demonstrated that treatment with Ad-RB94 induced an apoptosis rate of 26%. However, only a small number of positive cells were observed in the control and the Ad-LacZ-treated mice.

## Discussion

The results of the present study demonstrate that RB94 overexpression induces the G2/M cell cycle arrest of NSCLC *in vitro*. Overexpression of RB94 *in vivo* also induced the apoptosis of NSCLC cells and decreased the tumor cyclinB1 levels, which may cause G2/M phase block (12,13), and thereby inhibited the growth of the transplanted tumor.

It has been reported that RB94 is capable of inducing apoptosis in human pancreatic tumors and in head and neck cancer (6,8,10). Ad-RB94 transfection also induces cell cycle arrest in the G2/M phase and decreases telomerase activity in human head and neck squamous cell carcinoma (6). In the present study, the results demonstrated that Ad-RB94 transfection induced the G2/M phase block in the A549 lung cancer cells. The *in vivo* investigation also suggested that Ad-RB94 transfection decreased the levels of cyclinB1. The Cdk1/cyclinB1 complex plays a significant role in the progression of the cell cycle from the G2 to M phase, and downregulating cyclinB1 could cause G2/M phase block (12,13). Thus, these data indicated that the overexpression of Ad-RB94 may result in G2/M phase block in lung cancer cells. In the tumor xenograft study, the present study confirms that an adenoviral vector efficiently delivers the RB94 gene into targeted cells and suppresses the tumor cell growth. RB94 expression in the NSCLC tumor cells leads to apoptotic tumor cell death in the xenograft nude mouse model.

In conclusion, the present study provided evidence that RB94 significantly inhibits the growth of NSCLC cells *in vitro* and *in vivo*. These effects are elicited by arresting the cells at the G2/M phase and triggering apoptosis of the tumor cells. These results suggest that RB94 is a promising candidate for adjuvant therapy with radiation or chemotherapy, as tumor cells are most sensitive to radiation or cytotoxic drugs in this cell cycle phase. Further research is required to better elucidate the molecular pathways underlying these effects of RB94.
